# Charm Doctors

**Published:** 1861-11

**Authors:** 


"*Charm Doctors.*"—A correspondent in New England writes us that there are in his region a set of quacks styled "charm doctors," who sell charm receipts at from ten to twenty-five dollars each. He sends us the following as specimens:

1. *For the Bite of a Mad Dog.*—Say, lemus, lamus, remus, ramus, axilogue.

2. *Charm for those who are Mad—Man or Beast*—The hair being cut off, lay betony to the mould of the head, then write these words on a piece of cheese, antarbragan, tetragrammaton, and give it to the party so diseased.

3. *Charm for the Ague.*—Take a crust of bread and write on it in a legible hand the following words : Calinda, calindum, calindant, after which eat the crust.

4. *Charm for the Headache.*—A sure cure : Tie a halter about your head wherewith some one has been hanged. Our correspondent wickedly suggests that it be tied a little lower down—of course he refers to the seller of the charm.—*Medical and Surgical Reporter.*

